# Filtering Genes for Cluster and Network Analysis

**DOI:** 10.1186/1471-2105-10-193

**Published:** 2009-06-23

**Authors:** David Tritchler, Elena Parkhomenko, Joseph Beyene

**Affiliations:** 1 Department of Biostatistics, University of Toronto, Toronto, Ontario, Canada; 2 Department of Child Health Evaluative Sciences, Hospital for Sick Children Research Institute, Toronto, Ontario, Canada; 3 Department of Biostatistics, State University of New York at Buffalo, Buffalo, New York, USA; 4 Ontario Cancer Institute, Toronto, Ontario, Canada

## Abstract

**Background:**

Prior to cluster analysis or genetic network analysis it is customary to filter, or remove genes considered to be irrelevant from the set of genes to be analyzed. Often genes whose variation across samples is less than an arbitrary threshold value are deleted. This can improve interpretability and reduce bias.

**Results:**

This paper introduces modular models for representing network structure in order to study the relative effects of different filtering methods. We show that cluster analysis and principal components are strongly affected by filtering. Filtering methods intended specifically for cluster and network analysis are introduced and compared by simulating modular networks with known statistical properties. To study more realistic situations, we analyze simulated "real" data based on well-characterized E. coli and S. cerevisiae regulatory networks.

**Conclusion:**

The methods introduced apply very generally, to any similarity matrix describing gene expression. One of the proposed methods, SUMCOV, performed well for all models simulated.

## Background

Prior to cluster analysis or genetic network analysis it is customary, after initial quality screening, to remove genes considered to be substantively irrelevant from the set of genes to be analyzed. Typically genes which are informally judged to exhibit insufficient variation across samples are deleted. One obvious advantage of this is dimension reduction; clustering algorithms run faster and genetic network analysis may be simplified if there are fewer genes than samples. Another obvious advantage is clarity of interpretation: the biological meaning of a cluster or gene pathway is more easily discerned if the results of an analysis do not include irrelevant and distracting genes. We will demonstrate another ill effect of not filtering prior to analysis – bias in a cluster analysis of the data.

The accuracy and usefulness of a cluster analysis is strongly affected by the subset of genes to be analyzed, as determined by filtering the genes prior to the analysis. However, although much recent work has been devoted to the development of clustering methods, relatively little attention has been directed to the filtering step. This paper explores filtering in some depth and presents some new approaches. We establish the feasibility of data-based selection of genes, and its superiority to arbitrary thresholds.

## Results and Discussion

### The model

To investigate filtering we focus on the covariance structure of the data. Cluster and genetic network analysis focuses on the relatedness of the expression patterns of the genes being studied. We represent this by the covariance matrix of the gene expressions, and assume that there are groups of genes that are correlated among themselves while being uncorrelated with the other groups. There is a final set of genes *D *whose members are uncorrelated with each other and all other genes. It is this group of genes that we term irrelevant and wish to remove from the analysis.

This model implies a block structure for the appropriately ordered covariance matrix Σ. Suppose that *N *genes belong in the analysis and *d *genes are unrelated and should be deleted. Then

(1)

where Σ_*jj *_is a *n*_*j *_× *n*_*j *_within cluster covariance matrix, Δ is a *d *× *d *diagonal covariance matrix for the set *D *of irrelevant genes, and .

This model is an example of *modularity*, where a module is a part of an organism that is integrated with respect to a certain kind of process and relatively autonomous with respects to other parts of the organism. The modularity concept has gained popularity more-or-less simultaneously in molecular biology and systems biology, developmental biology and evolutionary biology, and cognitive psychology [1]. We assume that the within cluster covariance matrix Σ_*jj *_arises from an independent module which is a biological gene network. We consider two simple network architectures as examples in this paper.

#### SIMs

A SIM (Single Input Module) consists of a set genes that are controlled by a single transcription factor [2]. There is considerable experimental evidence that SIMs occur frequently [2,3]. For example, consider a SIM represented by the linear model for gene expression

(2)

(3)

where *β > *0 and the ϵ_*i *_are independent errors with mean 0 and variance 1. The covariance of all pairs of genes in this system is nonzero. The covariation among the *n*_1 _network genes is driven by the *hub y*_1 _which codes the transcription factor. We assume that genes not included in the SIM follow the model

(4)

where ϵ_*j *_is an independent error with mean 0 and variance 1. This will yield a covariance matrix of the form (1) with *m *= 1. The correlation of two non-hub genes is *β*^2^/(*β*^2 ^+ 1), and correlations with the hub are .

#### A causal chain

Another simple network architecture we consider is a causal chain of genes specified by the first-order autoregressive process

(5)

We assume that the process is stationary, whence

(6)

The correlation of expression between adjacent genes in the chain is *β*. We can regard *y*_1 _as coding the transcription factor which initiates the chain. The expression of genes outside the causal chain is distributed jointly as *iid N*(0, 1).

### The need for filtering

To investigate the necessity of filtering prior to cluster analysis, suppose that the set *D *of irrelevant genes is known. We will compare two strategies: 1) Preselection: filter out the set D and do a cluster analysis and 2) Postselection: do the cluster analysis and then delete the set *D *from the clusters. The final set of genes is the same, but the second method includes the irrelevant genes in the analysis phase. A data set of 50 arrays was generated using a multivariate normal distribution with five gene clusters each consisting of 40 genes. Within-cluster correlations of 0.40, between cluster correlation of zero, and variances of 1 defined the covariance matrix. The set *D *consisted of 400 genes each of which was independent of all others. We did a k-means cluster analysis (k = 5) of the genes for each of strategies 1) and 2) and compared the agreement of the respective gene clusters with the true grouping using the adjusted Rand index [4], which is a traditional criterion for assessment and comparison of different results provided by clustering algorithms. It is able to measure the quality of different partitions of a data set from a classification perspective, including partitions with different numbers of classes or clusters. The adjusted Rand index can range from 0 to 1, with 1 being perfect agreement. The data set was simulated 100 times and the average adjusted Rand index computed.

The means of the adjusted rand index for strategy 1 (Preselection) and for strategy 2 (Postselection) were 90% and 76%, respectively. Not deleting the irrelevant genes prior to cluster analysis introduces considerable bias. Thus even if an astute biologist has no trouble discarding irrelevant genes when presented with the results of an unfiltered cluster analysis, the validity of the results will be compromised. If we specify a sixth cluster when analyzing the larger set of genes, in the hope of isolating the irrelevant genes, the mean adjusted Rand index for strategy 2 rises to only 83%. Thus even if we knew the true number of clusters and added an "over flow" cluster to the analysis, postselection is inferior and the analysis is biased.

The same picture emerges if we are clustering arrays. A data set was generated using 25 arrays divided into 5 groups of 5. 40 genes had mean 1.3 for the first group of 5 arrays and mean 0 for the other arrays. Another 40 genes had mean 1.3 for the second group of 5 arrays and mean 0 for the other arrays, and so on for a total of 200 genes. Thus each group of arrays has a unique gene expression profile. Another set of 400 irrelevant genes was included which had mean zero for all the arrays. The genes had a multivariate normal distribution with the specified mean and the identity covariance matrix. We did a k-means cluster analysis of the arrays for each of strategies 1 and 2 and compared the agreement of the respective array clusters with the true grouping using the adjusted Rand index. This was replicated 100 times and the average adjusted Rand index computed.

**Figure 1:**
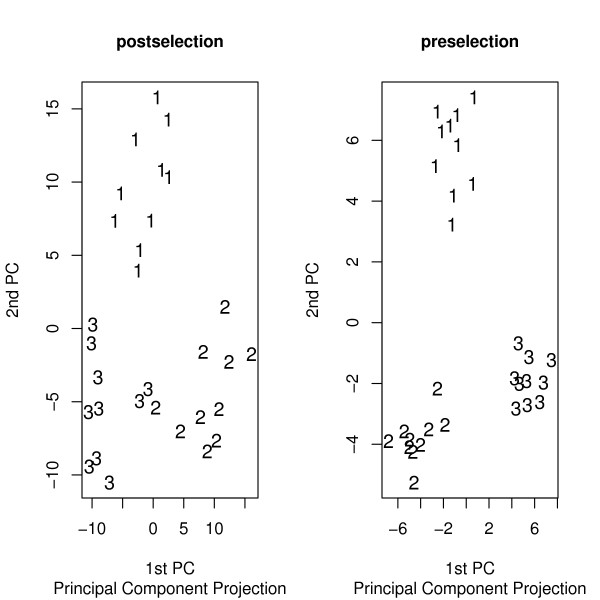
**Principal component plots**. In the left panel extraneous genes were deleted from the plot after the principal components were computed using all the genes. The right panel is for principal components computed from the relevant genes only.

The means of the adjusted Rand index for strategy 1 and for strategy 2 were 79% and 62%, respectively. Again, not deleting the irrelevant genes prior to cluster analysis of the arrays introduces considerable bias. Extraneous genes can also degrade more exploratory methods. A common exploratory method is to portray the arrays in two dimensions by projecting them onto the first two principal components. Figure 1 shows the principal component plots for arrays generated according to the preselection and postselection strategies already described. There were three groups of 10 arrays, each defined by a unique set of 40 genes over-expressed in that cluster. Group 1 was over-expressed for 40 genes and mean 0 otherwise. Group 2 was over-expressed for a different 40 genes and mean 0 otherwise, and likewise for Group 3. Another 1200 extraneous genes had mean 0 over all the arrays. For postselection the principal components were computed using all genes and then the irrelevant genes were deleted from the principal components plot. The preselection strategy computed the principal components using only the relevant genes. Figure 1 gives the principal component plots.

Filtering improves the informativeness of the principal component plot dramatically. The existence of bias in sample principal components in the presence of noise variables has been shown theoretically by [5]. The preceding has demonstrated that filtering irrelevant genes before analysis is desirable. We next investigate how this can be done.

### Motivation for proposed filtering methods

It is common to filter genes with small variance prior to clustering. The rationale is that genes which do not vary across samples contribute little information or may not be expressed, and genes which do not vary cannot covary. In this section we show that the pattern of covariance can be much more informative for filtering irrelevant genes. We will also demonstrate this later by simulation.

Consider the hub model given by equations (2) – (4). The variances of the genes in this network are



Note that the hub gene *y*_1 _which drives the underlying gene network has variance *σ*^2^, the same as the irrelevant genes . Clearly *y*_1 _should be included with the network genes in a cluster or inferred gene network, but filtering by a variance criterion would lump *y*_1 _with the irrelevant genes.

Now suppose that instead of filtering based on only the diagonal element of the covariance matrix, we associate with gene *i *the sum of the absolute values of the elements in row *i *of Σ, i.e. the summed absolute covariance with itself and the other genes. This measure is



Clearly the summed absolute covariance has more potential for filtering in the context of this network architecture. The measure differs more for the network versus non-network genes, and the hub *y*_1 _is now distinguished. We will refer to filtering based on the absolute row sums as covariance-based, and describe filtering based on small gene variance as variance-based.

**Figure 2:**
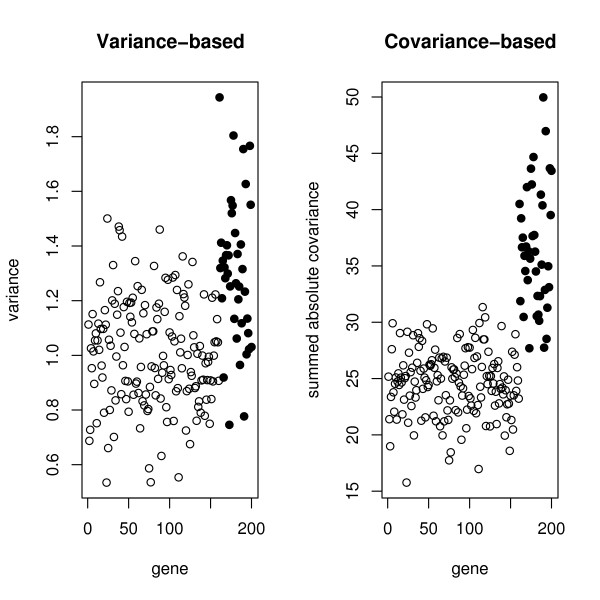
**Filtering criteria for a SIM**. The left panel shows the variances of the genes and the right panel shows the sum of absolute covariances. The dark points are the network genes.

To compare the two approaches, we generated data from a SIM of 40 genes, along with another 160 genes which were dormant in the process being studied. The regression coefficient was chosen so that the genes in the network had a correlation of .5 with the hub. There were 50 arrays. The left panel of Figure 2 shows the variances of the genes and the right panel shows the sum of absolute covariances. The dark points are the network genes. We observe that the network genes are more clearly distinguished using the absolute covariances.

Next we consider the causal chain architecture given by (5) and (6). For this stationary first-order autoregressive model each network variable has variance 1/(1 - *β*^2^) > 1 and the extraneous variables have variance 1, so the variance distinguishes the network genes. The sum of the absolute covariances for the *i*^*th *^network gene is



so this measure distinguishes the network genes more clearly that the variance.

The preceding results lead us to consider new filtering criteria which include off-diagonal elements of the covariance or correlation matrix.

### Filtering criteria

The first two new criteria we define are based on the covariance or the correlation.

• 

• 

where ∑ = {*σ*_*ij*_} and *R *= {*r*_*ij*_} are the covariance and correlation matrices, respectively.

The other two measures are motivated by the structure displayed in (1). The elements in a row of ∑ corresponding to a network variable are mixture of mean zero and mean nonzero random variables, indicating more variability than found in a row of non-network variables. We thus define two measures which exploit this property.

• *VARCOV*_*i *_= *Var*{|*σ*_*ij*_|; *j *≠ *i*}

• *VARCOR*_*i *_= *Var*{|*r*_*ij*_|; *j *≠ *i*}

where for a vector **x**, *Var*{**x**} is the sample variance of the sample **x**.

We will refer to filtering using only the variance as VAR.

To use the filtering criteria to identify the relevant genes, in this paper we use k-means clustering with k = 2 [6,7] to form two groups of genes based on their measured criterion value. The cluster of genes with the highest average criterion value are then taken to be relevant for cluster of network analysis.

### Simulations

To compare the filtering criteria, we simulated expression data using two models. Both models consisted of 5 independent equally-sized modules and a set of extraneous genes. There were 2000 genes in total, and we varied the percentage of extraneous genes from 50 per cent to 95 per cent, in increments of 5 per cent. For example, with 95 per cent extraneous genes, 1900 genes were extraneous and there were 5 modules consisting of 20 genes each. For 50 per cent, there were 1000 extraneous genes and each module consisted of 200 genes.

Model 1 – SIMs: The modules are SIMS. Within a SIM, the correlation between non-hub genes was .5.

Model 2 – Causal chains: The modules are causal chains. The correlation between adjacent genes in the chain was .7, so that a gene accounted for 49% of the variance of its successor in the chain.

Each model was generated 50 times, and the sensitivity and the positive predictive value was averaged over the 50 simulations for each of the criteria – variance, sumcov, sumcor, varcov, and varcor. The maximum possible standard deviation for any of the estimates was .007.

**Figure 3:**
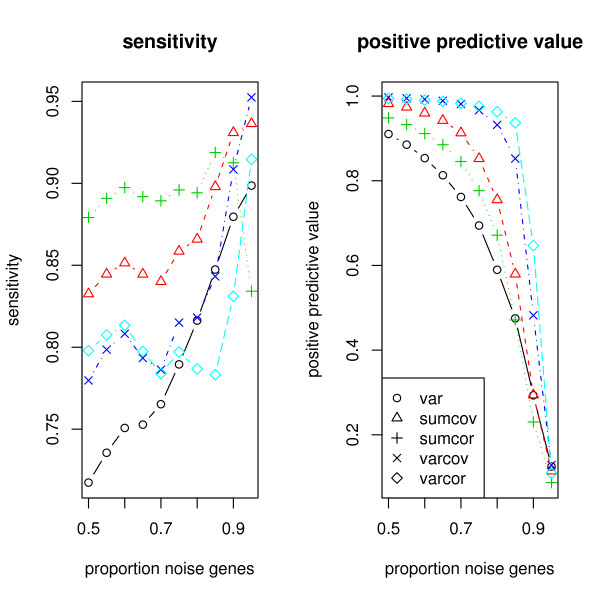
**Simulation of a network of SIMs**. The left panel shows the sensitivity achieved by the filtering methods and the right panel shows the positive predictive value.

For the Model 1 simulation of SIMs, Figure 3 shows the average sensitivity and average positive predictive value for each of the 5 criteria.

SUMCOR is the most sensitive measure when the percentage of noise genes is 80% or less, but is dominated by SUMCOV for higher percentages, and drops precipitously at 95% to be the worst of the measures. SUMCOV is more sensitive than VARCOR, VARCOV, VAR, with the marginal exception of VARCOV at 95%. In particular it is more sensitive than VAR throughout the range of percentages. The positive predictive value of all the methods deteriorates for high noise proportions, but VAR is the worst. The ranking of the methods with respect to positive predictive value is VARCOR, VARCOV, SUMCOV, SUMCOR, VAR. We conclude that VAR is not very informative for the SIM model, and SUMCOV and SUMCOR are good overall choices.

**Figure 4:**
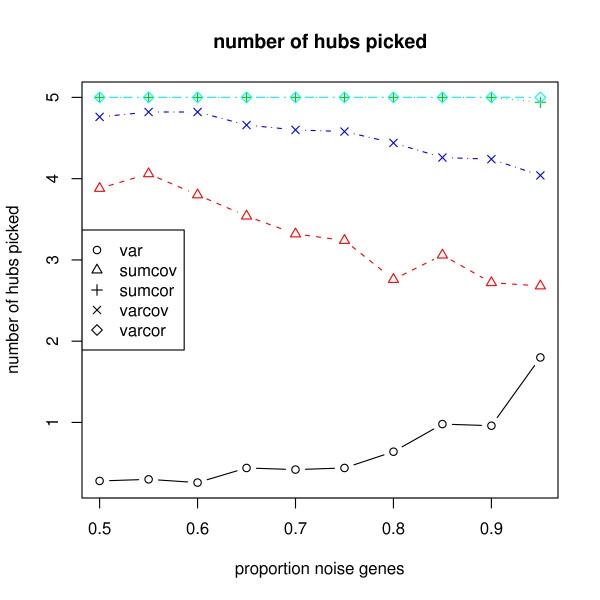
**Simulation of a network of SIMs**. The number of hub genes found by the filtering methods are displayed.

Figure 4 shows the number of hub genes found by the filtering methods.

As expected, VAR does very poorly at finding hub genes. The other methods are ranked as SUMCOR and VARCOR, VARCOV, SUMCOV, VAR. Both SUMCOV and VARCOV include aspects of VAR which is uninformative about hubs, and so probably pay a penalty for that reason.

**Figure 5:**
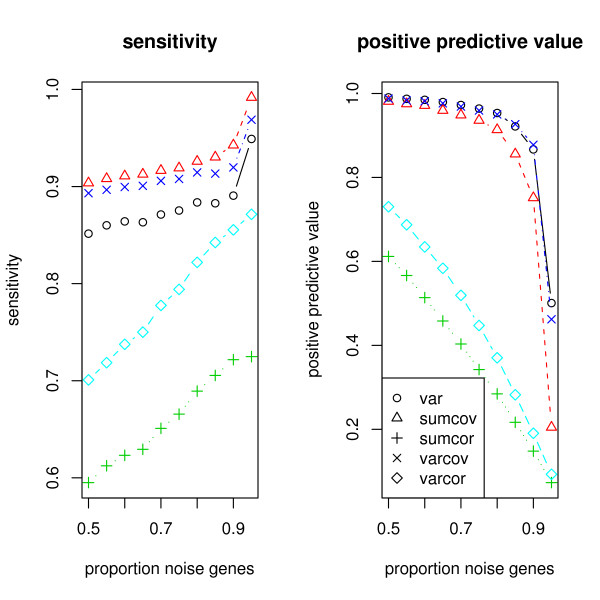
**Simulation of a network of causal chains**. The left panel shows the sensitivity achieved by the filtering methods and the right panel shows the positive predictive value.

For the Model 2 simulation of causal chains, Figure 5 shows the average sensitivity and average positive predictive value for each of the 5 criteria.

VAR does better for causal chains than for SIMs. This is because the off-diagonal covariances decay rapidly with increasing distance from the diagonal and have relatively little impact, so VAR captures most of the information. SUMCOV and VARCOV incorporate aspects of VAR, and have much better sensitivity and positive predictive value than SUMCOR and VARCOR which ignore the diagonal elements of the covariance matrix. Considering both sensitivity and positive predictive value, SUMCOV and VARCOV are competitive with VAR. SUMCOV has the advantage of also doing well for SIMs.

In practice, in the absence of knowledge of the underlying network architecture SUMCOV is a good overall choice, and is preferred to VAR since it captures additional structure. SUMCOR will perform well when VAR performs poorly. For example, when disparity of gene variances largely reflects experimental inconsistencies SUMCOR will benefit from not being a function of the variance (noise), while SUMCOV will be degraded. When the variance is informative, SUMCOV will include that information and also capture block structure, so it performs well for both SIMs and causal chains.

### A graphical method

As an alternative to the partitioning of the filtering criterion by k-means partitioning, we propose a graphical method. This is a q-q plot of the observed filtering criterion versus the criterion values for a null matrix of independent *N*(0, 1) random variables. We average 20 simulated null matrices to get a stable estimated of the null distribution. To use the plot we inspect it for a point of inflection corresponding to a slope change, and take that point as the threshold for the filtering criterion.

**Figure 6:**
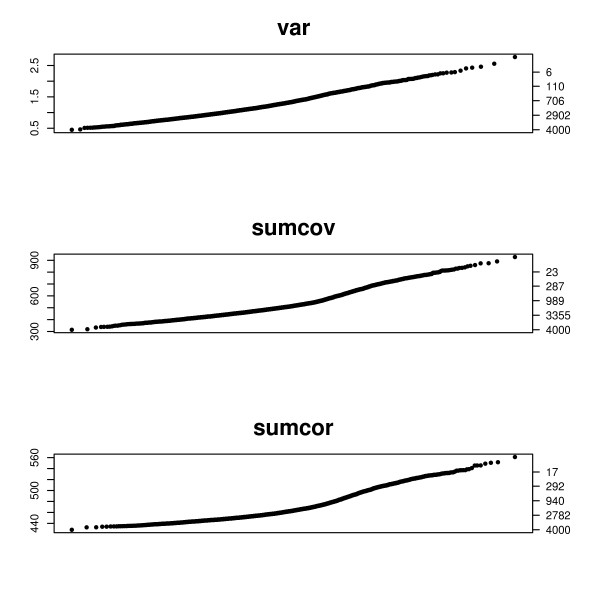
**q-q plots for a data set generated according to the SIM model**. q-q plots for a data set generated according to the SIM model. The left axis displays the criterion value, and the right axis gives the number of genes with criterion above that value. Half of the 2000 genes were network genes.

To demonstrate this we simulated a data set generated according to the SIM model used in the previous section. 1000 of the 4000 genes were network genes. Figure 6 shows the q-q plots for the criterions VAR, SUMCOV, and SUMCOR.

**Figure 7:**
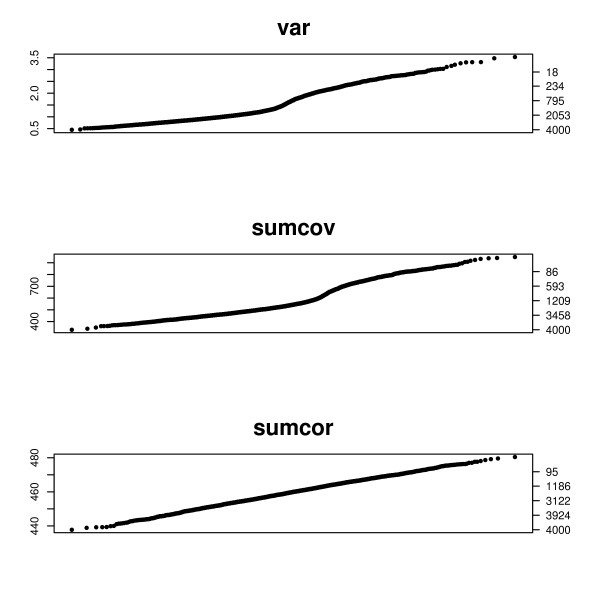
**q-q plots for the causal chain model**. q-q plots for a data set generated according to the causal chain model. The left axis displays the criterion value, and the right axis gives the number of genes with criterion above that value. Half of the 2000 genes were network genes.

The data for Figure 7 is similarly generated, using the causal chain model described in the previous section. The height of the plot is the value of the criterion, specified by the left vertical axis. The right vertical axis gives the number of genes with values greater than the corresponding height of the left axis. Thus one can read a cutoff value from the left axis and the consequent number of genes obtained on the right axis. In Figure 6 SUMCOV and SUMCOR show a clear inflection in their q-q plots. In both cases 1000 network genes is plausible from the plot. 2-means partitioning estimates 956 network genes based on SUMCOV and 1050 genes based on SUMCOR. The plot for VAR shows only a mild bend and offers little information as to a plausible cutoff. This is in agreement with the simulation results shown in Figure 5, where VAR did not do well for SIMs. 2-means partitioning of VAR suggests 1184 network genes.

In Figure 7 both the VAR and SUMCOV show a clear inflection in their q-q plots. In both cases 1000 network genes is plausible from the plot. 2-means partitioning estimates 975 network genes based on SUMCOV and 905 genes based on VAR. The plot for SUMCOR is straight and offers no information as to a plausible cutoff. This is in agreement with the simulation results shown in Figure 3, where SUMCOR performed poorly for the causal chain model. 2-means partitioning of SUMCOR suggests 2072 network genes.

### Analysis of E. coli and S. cerevisiae regulatory network data

The modular network models we have simulated certainly do not match the complexity of real microarray data. However, with real data the true underlying model is unknown so it is impossible to know which genes selected are true positives or misclassified irrelevant genes. To satisfy the criteria of realism and known properties, we generated datasets using SynTReN [8], a generator of synthetic gene expression data. This approach allows a quantitative assessment of the accuracy of the methods applied. The SynTReN generator generates a network topology by selecting subnetworks from the well characterized E. coli or S. cerevisiae regulatory networks. Then transition functions and their parameters are assigned to the edges in the network. Eventually, mRNA expression levels for the genes in the network are obtained by simulating equations based on Michaelis-Menten and Hill kinetics under different conditions. After the addition of noise, microarray gene expression measurements are produced.

We produced two synthetic expression datasets, one corresponding to the E. coli network topology and one for S. cerevisiae. In each dataset there were 100 network genes and 300 background genes. The 300 background genes have an underlying network structure, but are not perturbed and so propagate only error. This is a more realistic model for inactive genes than we simulated previously. We used the cluster addition option of SynTReN and set all parameters to their default values. We normalized the expression data produced using vsn [9].

**Figure 8:**
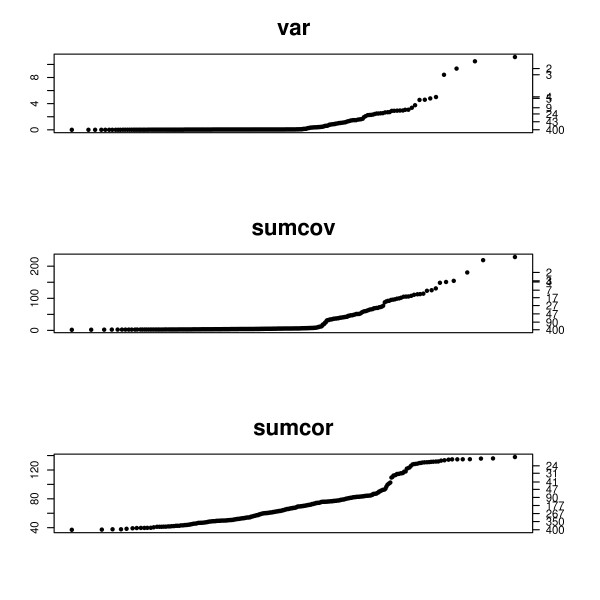
**q-q plots for the E. coli synthetic data**. q-q plots for the E. coli synthetic data. The left axis displays the criterion value, and the right axis gives the number of genes with criterion above that value.

Figure 8 shows the q-q plots for the E. coli synthetic dataset. Using k-means to partition the genes, the sensitivity/positive predictive value for the metrics is VAR: .3/1, SUMCOV: .45/1, and SUMCOR: .33/.66. VAR selected the 30 top genes and SUMCOV selected the top 45. This is discordant with the q-q plots and k-means clustering is obviously not partitioning the measurements well. The extremely high high measurements are apparently having too much influence. This leads us to try a more robust clustering method, PAM [7]. Using PAM, we obtain sensitivity/positive predictive value for the metrics VAR: .43/1, SUMCOV: .89/1, and SUMCOR: .33/.72. This analysis shows the value of the q-q plot for choosing a metric and partition. SUMCOR has a very clear inflection around 100 genes, and any partitioning should be in accord with this.

**Figure 9:**
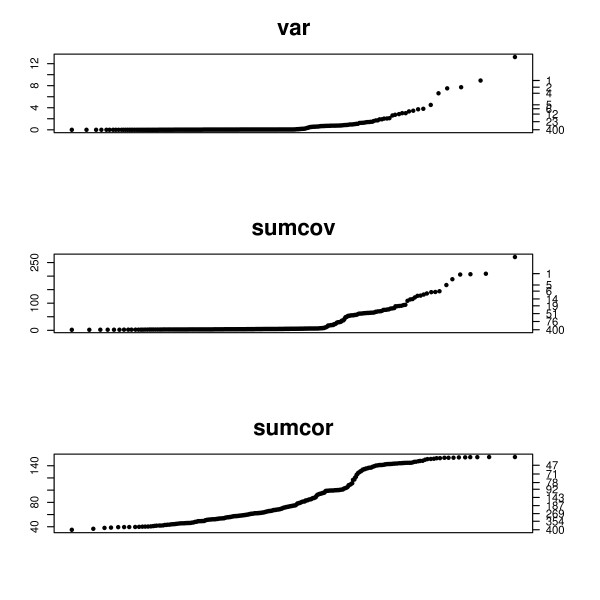
**q-q plots for the S. cerevisiae synthetic data**. q-q plots for the S. cerevisiae synthetic data. The left axis displays the criterion value, and the right axis gives the number of genes with criterion above that value.

Figure 9 shows the q-q plots for the S. cerevisiae synthetic dataset. Using k-means to partition the genes, the sensitivity/positive predictive value for the metrics is VAR: .06/1, SUMCOV: .71/1, and SUMCOR: .76/.56. This partitioning of VAR is discordant with the q-q plots and k-means clustering is obviously not partitioning the measurements well. As in the previous example the extremely high measurements are having too much influence. Using PAM, we obtain sensitivity/positive predictive value for the metrics VAR: .83/1, SUMCOV: .75/1, and SUMCOR: .42/.46. As before, the q-q plot is essential for choosing a metric and partition. SUMCOR has a very clear inflection around 100 genes, and any partitioning should be in accord with this.

SUMCOV partitioned well for both analyses, and had the most interpretable q-q plot with a clear inflection in the vicinity of the correct number of genes. VAR partitioned well for S. cerevisiae, suggesting that the connectivity may be less than for E. coli. The q-q plots for VAR were not very useful, which is a serious disadvantage for this metric. SUMCOR did not partition well for either dataset, but the q-q plots for this metric were very informative.

## Conclusion

This paper presents objective methods for evaluating and choosing metrics and thresholds for filtering genes prior to cluster and network analysis. We introduced a model for genetic data with modular network structure and a substantial proportion of irrelevant "noise" genes. We considered two variations, SIMS and causal chains. The examination of these models shows that the common practice of filtering out genes with low variance can be justified for some models, and introduces alternatives which are superior for other models and conditions.

Real biological phenomena are certainly more complex than the simple models we considered, and probably represent a mixture of complex architectures. We have shown that using VAR can miss hubs, so that metric may be considered to be biased against hubs. Variables with higher connectivity will be more easily detected using SUMCOV and SUMCOR. SIMs have a stronger block-diagonal structure, but the connectivity is still rather limited and more complex structures may be expected to have larger off-diagonal entries. In general, variables with higher connectivity will be more easily selected by the new metrics SUMCOV and SUMCOR. An advantage of SUMCOV is the incorporation of both diagonal and off-diagonal elements of the covariance matrix. However, if the diagonal elements reflect experimental inconsistency this becomes a disadvantage. We advocate using our graphical approach to chose the most informative measure.

We adopted 2-group clustering algorithms to classify genes because these are familiar methods for identifying the components of a mixture of two distributions. However, we don't claim any inherent advantage for this approach. Further investigation of mixture methods in the context of varying types of data could be done, but the intent of this paper is to point out the feasibility of data-based selection of genes, and its superiority to arbitrary thresholds. The analysis of the E. coli and S. cerevisiae shows that in a realistic setting robustness is an important consideration when selecting a partitioning method.

Although we have restricted our discussion to measures based on the covariance matrix, the ideas are much more general. The covariance could be replaced by any similarity measure and analogous measures computed. Self-similarity would be substituted for VAR, row sums of the similarity matrix for SUMCOV, and row sums of the off-diagonal similarities (after normalization by self-similarity) for SUMCOR. The q-q plot could again be used to judge relative efficacy.

Methods have been developed recently which simultaneously cluster and perform variable selection [10,11]. These methods are restricted to model-based clustering and will not apply to other clustering algorithms, varied definitions of gene similarity, or other network inference methods. An advantage of our method is that it is model-free and can be used in conjunction with any clustering algorithm or similarity matrix. We recommend that other information relevant to filtering be employed before focusing on the covariance matrix. For example, conventionally genes considered to be unexpressed based on the small magnitude of their gene expression measurements are deleted, and such screening should be performed prior to applying the methods of this paper.

## Authors' contributions

DT, EP and JB contributed equally to methods development. EP and DT performed the computer simulations. DT analyzed the example data sets. All authors read and approved the final manuscript.
